# Preparation of TiO_2_-Decorated Boron Particles by Wet Ball Milling and their Photoelectrochemical Hydrogen and Oxygen Evolution Reactions

**DOI:** 10.3390/ma9121012

**Published:** 2016-12-14

**Authors:** Hye Jin Jung, Kyusuk Nam, Hong-Gye Sung, Hyung Soo Hyun, Youngku Sohn, Weon Gyu Shin

**Affiliations:** 1 Department of Mechanical Engineering, Chungnam National University, Daejeon 34134, Korea; gpwlsdlsz@naver.com; 2 Department of Chemistry, Yeugnam University, Gyeongsan 38541, Korea; zxvcod@naver.com; 3 School of Aerospace and Mechanical Engineering, Korea Aerospace University, Goyang, Gyeonggi-do 21071, Korea; hgsung@kau.ac.kr; 4 The Fourth R&D Institute, Agency for Defense Development, Daejeon 34188, Korea; hshyunin@add.re.kr

**Keywords:** boron particle, wet ball milling, TiO_2_ coating, photoelectrochemical, hydrogen evolution, oxygen evolution

## Abstract

TiO_2_-coated boron particles were prepared by a wet ball milling method, with the particle size distribution and average particle size being easily controlled by varying the milling operation time. Based on the results from X-ray photoelectron spectroscopy, transmission electron microscopy, energy dispersive X-ray analysis, and Fourier transform infrared spectroscopy, it was confirmed that the initial oxide layer on the boron particles surface was removed by the wet milling process, and that a new B–O–Ti bond was formed on the boron surface. The uniform TiO_2_ layer on the 150 nm boron particles was estimated to be 10 nm thick. Based on linear sweep voltammetry, cyclic voltammetry, current-time amperometry, and electrochemical impedance analyses, the potential for the application of TiO_2_-coated boron particles as a photoelectrochemical catalyst was demonstrated. A current of 250 μA was obtained at a potential of 0.5 V for hydrogen evolution, with an onset potential near to 0.0 V. Finally, a current of 220 μA was obtained at a potential of 1.0 V for oxygen evolution.

## 1. Introduction

Boron has been employed as an energetic material for ramjet solid additives and for solid propellants of ducted rockets because of its exceptionally high combustion enthalpy per unit volume [1,2,3]. Boron has also been used as a catalyst additive and support material [4,5,6,7,8,9]. Aramendia et al. prepared MgO-B_2_O_3_ mixed oxides and found an increase in selectivity to dehydrogenation upon increasing the boron content in the oxide catalyst [6]. In addition, the catalytic activity of alumina-boria catalysts supported on porous or non-porous alumina was shown to increase selectivity in the oxidation of ethane to ethylene; this was due to an increase in acidity upon the addition of boron oxide [7]. Furthermore, Shin and co-workers used boron particles as a CeO_2_ catalyst support material based on dry and wet ball milling methods, and subsequently examined the CO oxidation activity of the resulting particles. They found that boron-CeO_2_ hybrid materials showed enhanced catalytic activity compared with naked boron and CeO_2_ nanoparticles [8].

Titanium is one of the most widely studied and applied catalyst materials in reactions such as water splitting (hydrogen and oxygen evolution reactions) and CO_2_ reduction [10,11,12,13,14,15,16,17,18], with the hybridization of two or more materials being employed to increase catalytic activity [17,18,19,20,21,22,23,24]. Roy et al. prepared transition metal (Fe, Co, and Cu)-doped TiO_2_ nanocrystals and examined their electrochemical oxygen evolution reactions (OERs) [17]. For the OER of metal-doped TiO_2_, they achieved a lower potential of ~1.12 V, which was 0.33 eV lower than that for undoped TiO_2_. This enhancement was attributed to a change in the electronic band position [17]. In addition, Xiang et al. prepared a TiO_2_/MoS_2_/graphene hybrid, which exhibited a dramatic increase in H_2_ production rate with a quantum efficiency of 9.7% at 365 nm [20]. Furthermore, Hu et al. coated Si, GaAs, and GaP photoanodes with TiO_2_ by atomic layer deposition, which prevented corrosion and resulted in continuous O_2_ evolution in a 0.1 M KOH solution at photocurrent densities >30 mA·cm^−1^ and ~100% Faradaic efficiency [21]. Moreover, Hou et al. reported that mesoporous TiO_2_/CuO/Cu materials exhibit enhanced photocatalytic H_2_ evolution (3.5× higher) compared to commercial mixed phase TiO_2_ (Degussa, P25) [22]. Additionally, Terashima reported a p-n heterojunction photoelectrode of boron-doped diamond(p-type)/TiO_2_(n-type) prepared by microwave plasma chemical vapor deposition followed by sputter coating [23]. They observed a 1.6-fold increase in photoelectrochemical performance, compared with that of bare TiO_2_, which was attributed to charge carrier separation at the interface. Finally, Zhao et al. synthesized B/N-co-doped TiO_2_ by the thermal treatment of B-doped TiO_2_ with urea, where B-TiO_2_ was previously prepared via hydrothermal methods [24]. They observed an increase in photocatalytic activity both under UV and visible light, attributed to the formation of Ti^3+^ following B doping (B + Ti^4+^ → 1/n·B^δ+^ + Ti^3+^) and B–N bond formation. Ball-milling method has also effectively been used to synthesize large-scale boron nitride nanotubes (BNNT) [25,26,27]. Li et al. first mixed ball-milled nano-size boron particles with metal nitrate in ethanol, painted (sprayed or printed) on a substrate, and then annealed in a nitrogen condition to obtain BNNT films [25,26,27].

We therefore chose to investigate boron particles as a catalyst support material in addition to the development of a novel method to synthesize TiO_2_-coated boron particles via a wet milling process using titanium isopropoxide (TTIP) as the coating material. The advantage of using wet ball milling process compared to dry milling process is that it is possible to make a more uniform coating on the surface of particles by decomposing the precursor liquid and forming chemical bonds on the surface of particles using high energy in the ball milling process [8,28,29,30]. We also investigated the physicochemical properties of the TiO_2_-coated boron particles using transmission electron microscopy (TEM), X-ray diffraction (XRD), scanning electron microscopy (SEM), nanoparticle size analysis (NPSA), X-ray photoelectron spectrometry (XPS), and Fourier transform infrared (FT-IR) spectroscopy. Furthermore, we examined the potential application of TiO_2_-coated boron particles to photoelectrochemical hydrogen and oxygen evolution reactions (HERs and OERs) to meet current energy and environmental needs.

## 2. Results and Discussion

**Figure 1:**
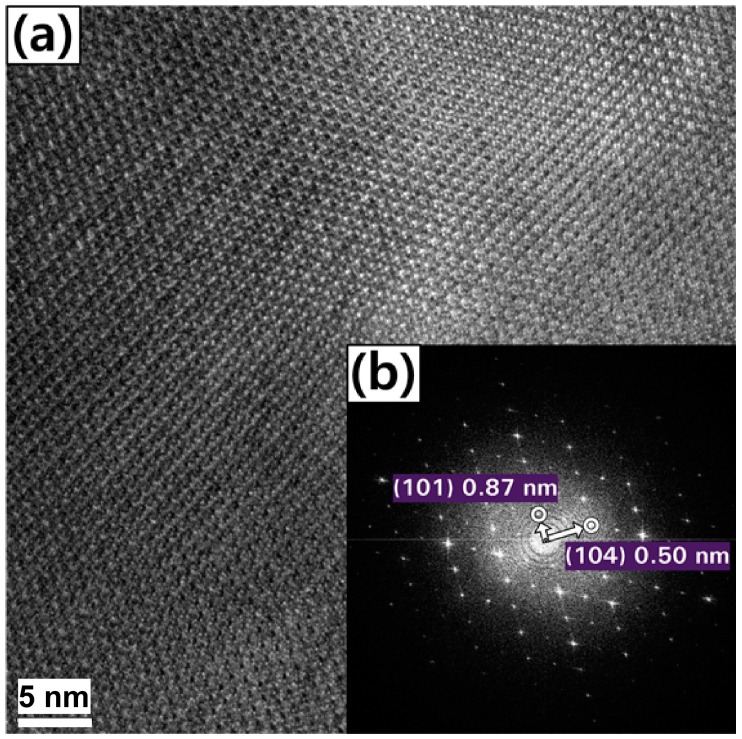
Transition electron microscopy (TEM) images of an un-milled boron particle: (**a**) The high-resolution TEM image, and (**b**) the corresponding fast Fourier transformation (FFT) pattern.

Un-milled boron particles were characterized by TEM (Figure 1). Figure 1a,b shows the high-resolution TEM image of an un-milled boron particle and the fast Fourier transformation (FFT) pattern of the image, respectively [28]. Indeed, the FFT patterns show a highly crystalline nature, and the calculated spacings of 0.87 nm and 0.50 nm were consistent with the (101) and (104) planes of the rhombohedral crystal structure of boron, respectively.

**Figure 2:**
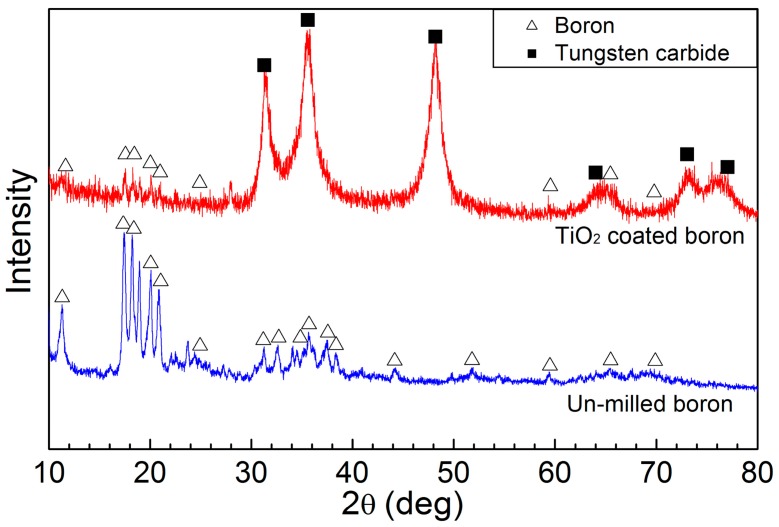
X-ray diffraction (XRD) patterns of the 8 h-wet milled TiO_2_-coated boron particles (**top**) and un-milled boron particles (**bottom**).

Figure 2 shows the XRD pattern of the boron particles before (bottom) and after (top) the wet milling process. The XRD pattern of the un-milled boron particles indicated a rhombohedral crystal structure (Joint Committee on Powder Diffraction Standards, JCPDS 71-0157). In contrast, in the wet milled boron sample, tungsten carbide from the milling jar was present as an impurity, and so an XRD pattern corresponding to the WC phase was also observed (JCPDS 72-0097) [8,28]. No trace of TiO_2_ was observed by XRD, indicating that TiO_2_ was present in an amorphous form. TiO_2_ was believed to be formed by hydration reaction (calculated conversion yield of ~5%) of TTIP with residual moisture in the nitrogen condition.

**Figure 3:**
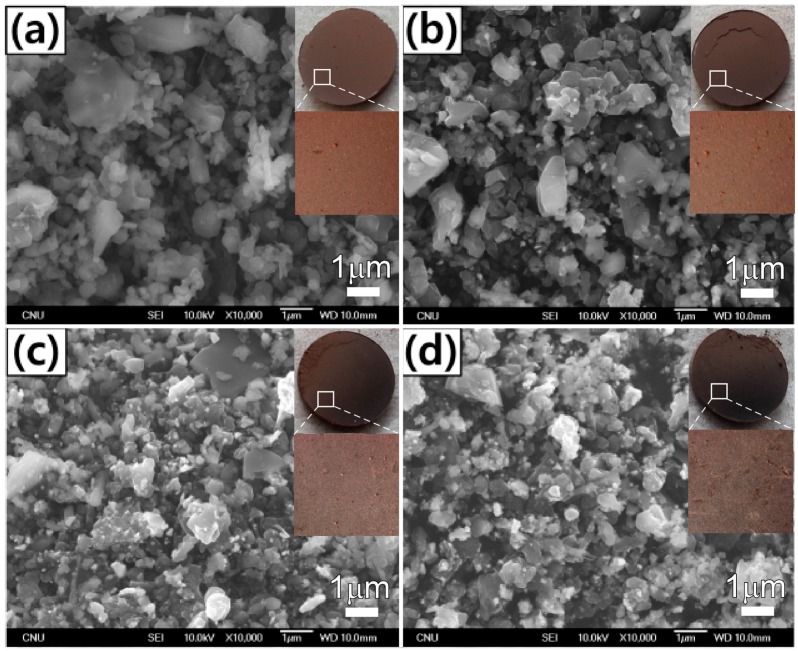
Scanning electron microscopy (SEM) images of the wet milled boron particles with milling operation times of: (**a**) 0 h; (**b**) 2 h; (**c**) 4 h; and (**d**) 8 h. The insets show the corresponding photograph and optical microscope images of the pelletized samples.

Figure 3 shows the SEM images of the boron particles wet milled with TTIP over milling times of 0 h, 2 h, 4 h, and 8 h. Figure 3a (0 h) shows that the average boron particle size was 800 nm, as noted in the specification provided by the manufacturer. As shown in the series of images, the particles became smaller and rougher as the milling operation time increased. In addition, the corresponding photograph and optical microscope images show that the brown color became gradually darker with an increase in milling time. The milled boron samples were analyzed by scanning electron microscopy/energy-dispersive X-ray spectroscopy (SEM-EDX). The atomic fractions of Ti and W were increased with increasing ball-milling times (Table 1). For the 8 h wet-milled boron samples, the atomic fractions of B, O, Ti, and W were observed to be 92.75%, 4.85%, 0.35%, and 2.05%, respectively.

**Figure 4:**
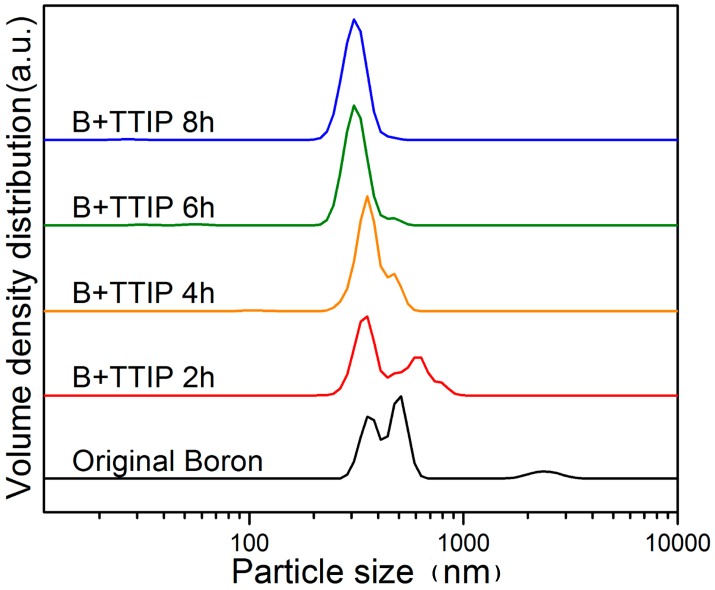
Particle size distributions of bare boron and B + titanium isopropoxide (TTIP) (2, 4, 6, and 8 h) samples measured by nanoparticle size analysis (NPSA).

The size distributions of the wet milled boron particles obtained following various milling times were then measured by NPSA [28] (Figure 4). To obtain accurate size distributions, several spectra were recorded for each sample and an average was taken. The size distribution of the un-milled boron particles was mainly in the region of 300–600 nm, although some particles were observed in the region between 2 and 3 μm. Based on NPSA data, the average size of the un-milled boron particles was calculated to be 600 nm. However, upon wet milling the samples for 2, 4, 6, and 8 h, the average sizes of the boron particles decreased to approximately 453 nm, 373 nm, 316 nm and 312 nm, respectively. In addition, a very small fraction (0.01%–0.2%) of particles with sizes in the range of tens of nm was observed for all samples. Moreover, the 4 h, 6 h, and 8 h samples contained small fractions of particles in the size ranges of 72–149 nm, 24–72 nm, and 21–32 nm, respectively, although the peaks corresponding to these small particles were not clearly observed in the size distribution plots (Figure 4). These results confirmed that overall, the size of the wet milled boron particles decreased upon increasing the milling operation time, with greater reductions being observed for larger particles. This resulted in the size distribution becoming narrower with an increase in the milling operation time.

**Figure 5:**
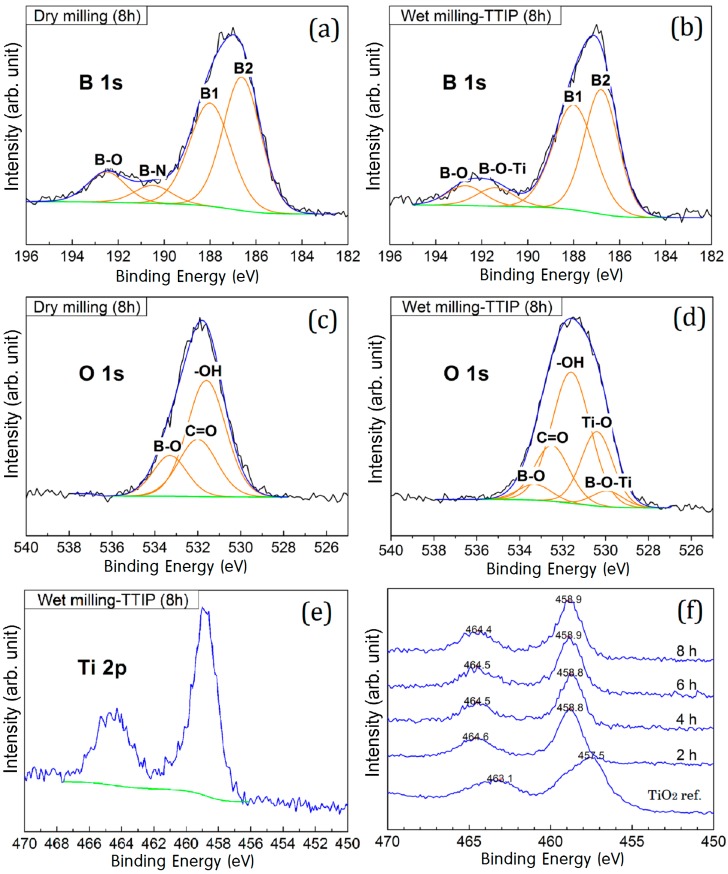
High-resolution narrow scan X-ray photoelectron spectra (XPS): (**a**) B 1s spectrum of the 8 h dry milled boron sample; (**b**) B 1s spectrum of the 8 h wet milled boron sample; (**c**) O 1s spectrum of the 8 h dry milled boron sample; (**d**) O 1s spectrum of the 8 h wet milled boron sample; (**e**) Ti 2p spectrum of the 8 h wet milled boron sample; and (**f**) Ti 2p spectra of the pure TiO_2_ (rutile phase) for 2 h, 4 h, 6 h, and 8 h wet milled boron samples.

Figure 5a–e shows the XPS spectra of the boron samples following either dry or wet milling for 8 h. Each XPS spectrum was corrected using a reference C 1s peak at 284.5 eV. Figure 5a,b shows the B 1s XPS spectra for the dry and wet milled samples. For the dry milled sample (Figure 5a), B 1s XPS binding energy peaks were identified at 186–194 eV, with four different chemical states of boron being identified. The major peaks, located at 186.8 and 188.0 eV, were assigned to elemental boron (B–B) [3], while the smaller broader peaks were assigned to the B–O and B–N species, which exhibit binding energies of 192.5 eV and 190.5 eV, respectively. It is likely that the B–N species was formed by a reaction between the naked surface of the boron particles and nitrogen gas. In contrast, for the wet milled sample (Figure 5b), the small peak could be assigned to B–O and B–O–Ti species with binding energies of 192.5 eV and 191.4 eV, respectively. Moreover, the lower intensity of the B–O signal in the wet milled sample was likely due to the formation of B–O–Ti bonds on the surface of the wet milled sample [31,32,33]. A thinner boron oxide layer was reported to be present on the wet milled boron particles compared to the dry milled boron particles at a fixed operation time [33].

As indicated above, Figure 5c,d shows the O 1s XPS spectra of the dry and wet milled boron samples, respectively. As shown in Figure 5c (the dry milled sample), the O 1s region showed peaks at 531.6 eV, 532.5 eV, and 533.3 eV, which correlated to hydroxyl groups (-OH), C=O species formed by the reaction between boron and tungsten carbide, and boron oxide (B–O). In contrast, the O 1s spectrum of the wet milled boron sample (Figure 5b) was broader due to the presence of signals corresponding to Ti–O species at 530.4 eV and B–O–Ti species at 529.9 eV [33,34,35]. In addition, Figure 5e shows the Ti 2p XPS spectrum of the 8 h-wet milled boron samples. The binding energies of Ti 2p_3/2_ and Ti 2p_1/2_ were observed at 464.5 eV and 458.8 eV, which could both be attributed to Ti^4+^ (TiO_2_) [14,18,36,37]. Furthermore, Figure 5f shows the Ti 2p XPS spectra of the various wet milled boron samples in addition to that of a pure TiO_2_ sample. For all wet milled samples, signals of the Ti 2p XPS spectra were observed at higher binding energies (i.e., 0.8 eV higher) compared with those of the reference TiO_2_ sample. This suggests that the overlayer Ti atoms show different chemical states on the boron surface, compared with the reference sample.

The surface compositions and chemical states of the various samples were then analyzed by XPS as summarized in Table 2. Prior to sputtering, the atomic fraction of oxygen was 23% for the un-milled boron sample. However, this value decreased with increasing sputtering time, indicating that oxygen is present in the form of a boron oxide (B_2_O_3_) layer on the boron surface. For the wet milled boron samples, an increase in ball-milling time before sputter time resulted in increases in the atomic fractions of Ti and O from 1.42% and 3.59% to 25.10% and 33.37%, respectively. This was attributed to an increase in the thickness of Ti–O layer with increasing ball-milling time. In addition, low levels of W, Co, and F impurities were detected, likely originating from tungsten carbide milling jar and ball (W and Co), and the un-milled boron (F). The W impurity level was also increased with increasing ball-milling time.

**Figure 6:**
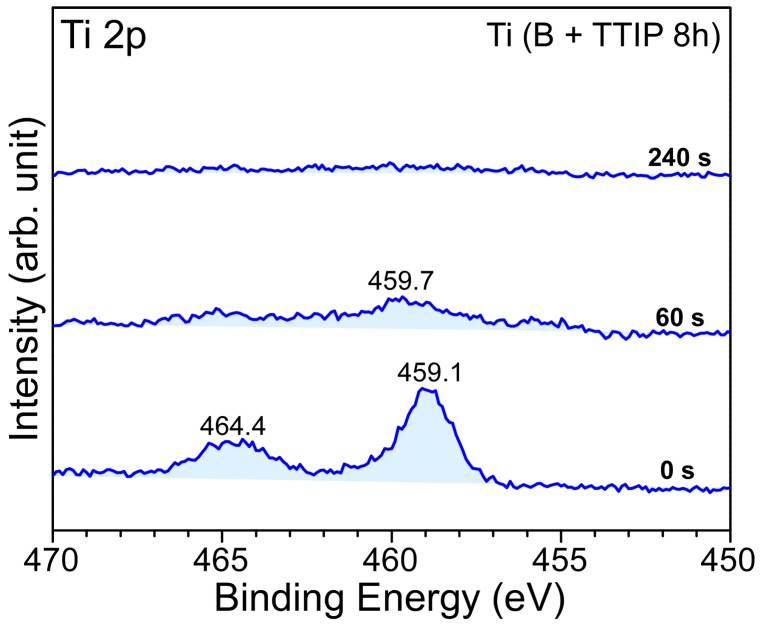
Variation in the Ti 2p XPS spectra with increasing sputtering time for the 8 h wet-milled boron sample.

Sputtering was performed using an Ar^+^ ion beam in the XPS instrument [18,38]. Figure 6 shows the high resolution Ti 2p XPS spectra of the 8 h wet-milled samples with sputtering times of 0 s, 60 s, and 240 s. The intensity of Ti 2p signals dramatically decreased with 60 s sputtering, with all signals disappearing after 240 s. This confirmed that the titanium component existed as a thin layer on the boron surface. The W impurity was still present after the 240 s of sputtering. This reflects that some bigger W particles were present on the boron surface and were not completely removed by the sputtering (will be further discussed later).

**Figure 7:**
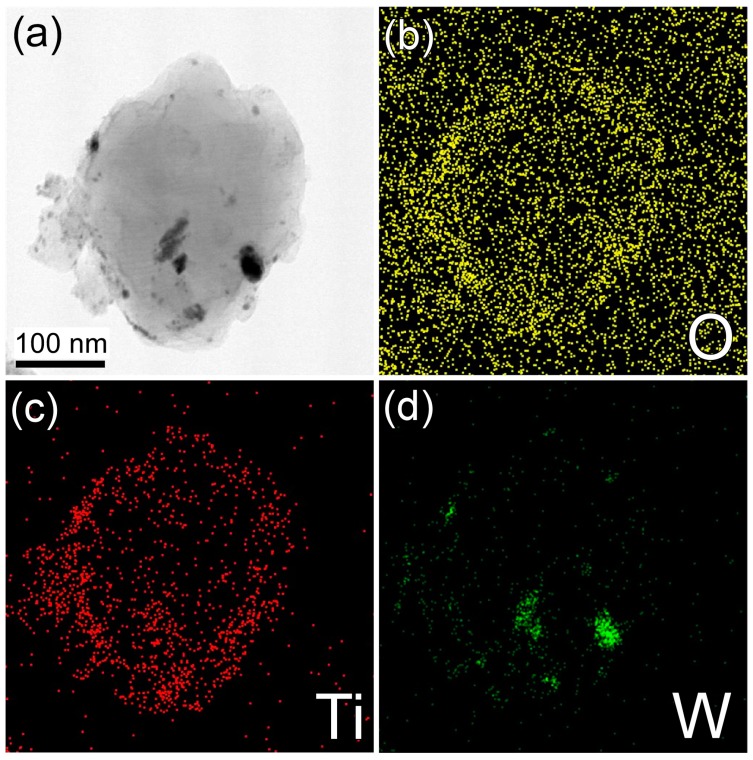
TEM image and energy-dispersive X-ray spectroscopy (EDX) results: (**a**) White field image of an 8 h wet-milled boron particle, and elemental mappings of (**b**) O; (**c**) Ti; and (**d**) W.

The 8 h wet-milled boron sample was then analyzed by transmission electron microscopy/energy-dispersive X-ray spectroscopy (TEM-EDX) to yield information regarding the morphology of titanium on the boron surface. Figure 7a shows the white field image of the mapped particles, while the elemental mapping results for O, Ti, and W are given in Figure 7b–d, respectively. As shown in Figure 7b, O was present both on the particles and on the TEM grid, but was more abundant on the boron particle surface. In contrast, Ti was mainly observed on the boron surface (Figure 7c). Accordingly, it was confirmed that the boron particle surface was covered with a material containing both O and Ti. Finally, as shown in Figure 7d, the distribution of W originating from tungsten carbide in the milling jar is consistent with the position of the dark spots shown in Figure 7a. Based on the black-colored particles in Figure 7a and green-colored regions of Figure 7d, we believe that the particles on the bigger boron particle are mainly due to W.

**Figure 8:**
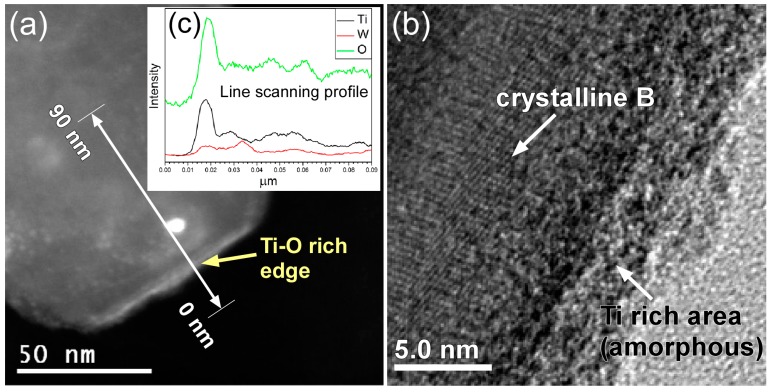
Analysis of an 8 h wet-milled boron particle: (**a**) Scanning transmission electron microscopy/high-angle annular dark field (STEM/HAADF) image. EDX line scanning analysis was performed as indicated by the green line; (**b**) High resolution TEM image of the particle; and (**c**) STEM/EDX line scanning results for the 8 h wet-milled boron particles.

The chemical composition of the wet milled boron particles was obtained via EDX line scanning using scanning transmission electron microscopy (STEM) and high-angle annular dark field (HAADF) images. Figure 8a shows the STEM image of a wet milled boron particle of ~150 nm diameter, where the green line indicates where line scanning was performed. Figure 8b shows the corresponding high resolution TEM image for the surface of the wet milled boron particle, which indicated an amorphous structure on the thin Ti–O rich edge, despite a crystal structure of boron being observed in the other region. Comparison with the XRD pattern shown in Figure 2 therefore confirmed that amorphous TiO_2_ exists on the wet milled boron particle. Finally, as shown in Figure 8c, the EDX results indicate a TiO_2_ layer thickness of ~10 nm.

**Figure 9:**
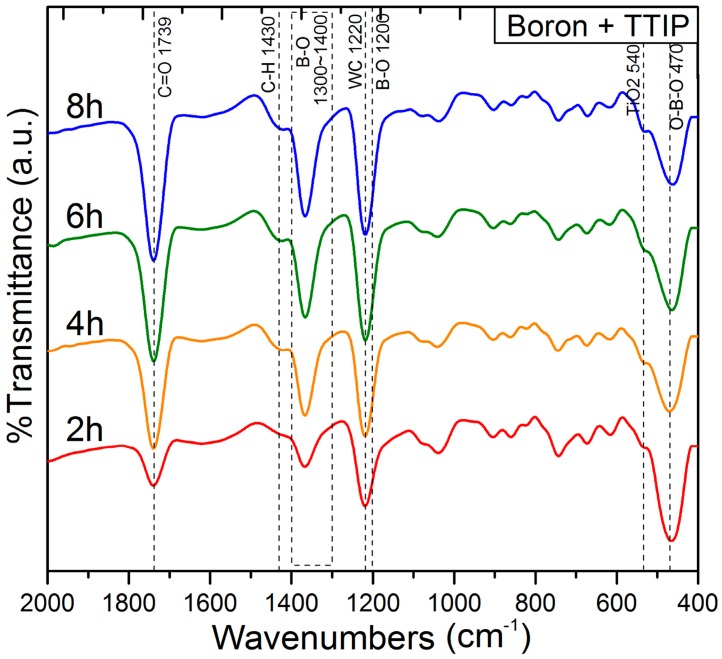
Variation in the Fourier transform infrared (FT-IR) spectra of the samples in the presence of TTIP with increasing milling operation time.

Figure 9 shows the FT-IR spectra of the wet milled boron particles recorded after a range of milling times in the presence of TTIP. Upon increasing the milling operation time, the intensities of the TiO_2_ and B–O peaks at 540 cm^−1^ and 1300–1400 cm^−1^ increased due to an increase in the amount of B–O–Ti on the surface of the boron particle [39,40]. In contrast, the intensity of the O–B–O peak at 470 cm^−1^ decreased with higher milling times, indicating that B_2_O_3_, i.e., the oxide layer on the boron surface, was removed during the milling process. The intensities of the remaining two peaks at 1220 cm^−1^ and 1536 cm^−1^, which were attributed to WC and C=O [41], respectively also increased upon increasing the milling time due to increased contamination during the milling process.

**Figure 10:**
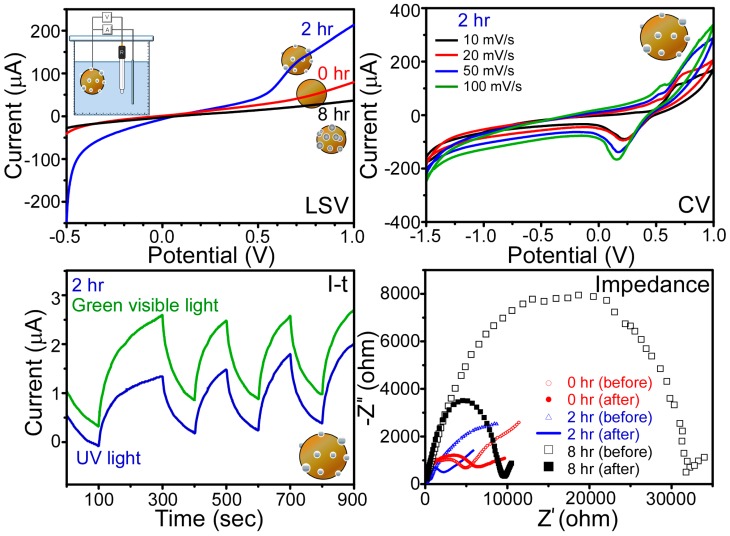
Linear sweep voltammograms (LSVs), cyclic voltammograms (CVs), and current-time (I-t) amperometry curves under cyclic ON and OFF light conditions at 0.3 V, and impedance curves for selected bare boron, B@Ti (2 h), and B@Ti (8 h) samples.

Finally, the linear sweep voltammograms (LSVs), cyclic voltammograms (CVs), current-time (I-t) amperometry curves, and impedance curves for the various samples were recorded and are shown in Figure 10 [42,43,44]. These results allow a preliminary examination of the potential applications of the Ti-decorated boron particles in photoelectrochemical HERs and OERs. For the LSVs recorded between −0.5 and 1.0 V, the current intensity was dependent on the sample, giving an order of B@Ti (8 h) < bare B << B@Ti (2 h), where the B@Ti (2 h) sample exhibited the highest catalytic activity. For the HER, a current density of 250 μA was obtained at a potential of 0.5 V, and the HER began to take place at a very low potential, i.e., close to 0.0 V. In contrast, the onset potentials for the B@Ti (8 h) and bare B samples were >0.5 V. For the OER of the B@Ti (2 h) sample, a current density of 220 μA was obtained at a potential of 1.0 V. In addition, the OER of the B@Ti (2 h) sample began to increase sharply at ~0.5 V. These observations indicated that the B@Ti (2 h) sample exhibited a dramatic enhancement in both the HER and OER compared to bare B. For the poorer HER and OER performance for B@Ti (8 h), the increased WC impurity may more significantly negate the performance. The WC impurity was found to be increased (in Table 2) as the ball-milling time was increased. The CVs obtained for the B@Ti (2 h) sample at scan rates of 10 mV·s^−1^, 20 mV·s^−1^, 50 mV·s^−1^ and 100 mV·s^−1^ are also shown in Figure 10. The corresponding CVs of the bare B and B@Ti (8 h) samples exhibited significantly lower current intensities, consistent with the LSVs. Moreover, the distance between the oxidation and reduction peaks became wider and the peak intensities increased with increasing scan rates. Subsequently, the photocurrent response of the B@Ti (2 h) sample was qualitatively examined using current-time (I-t) amperometry, with the sample exhibiting a photocurrent response under both cyclic ON and OFF UV (365 nm) and visible light (532 nm) irradiations at an applied potential of 0.3 V versus Ag/AgCl reference electrode. This resulted in the sample exhibiting photocatalytic hydrogen evolution and oxygen evolution activities by electron and hole generation upon the absorption of light [14,15,16,36]. For the real (Z′) and imaginary (Z″) parts of the impedance Nyquist plots (Figure 10), semi-circles were observed in the high frequency region with the sizes following the order B@Ti (8 h) > bare B > B@Ti (2 h) [37,38,39], where smaller semi-circles correspond to higher currents. Indeed, in all cases, the semi-circles decreased in size following the measurements, indicating an enhancement in current by reduction in the interfacial charge transfer resistance.

## 3. Materials and Methods

### 3.1. Metallic Oxide Coating on Boron by Wet Ball Milling

**Figure 11:**
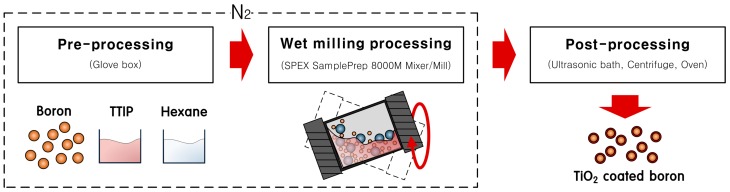
Schematic diagram of the wet ball milling process.

Boron particles were subjected to wet ball milling using a SPEX SamplePrep 8000M Mixer/Mill. A schematic diagram of the experimental set-up employed for the wet milling process is shown in Figure 11. Tungsten carbide balls have the diameter of 5 mm. The rotational speed of a motor in SPEX Sample Prep 8000M Mixer/Mill was 1725 RPM @ 60 Hz. Inside a nitrogen-filled glove box, a tungsten carbide milling jar was filled with boron powder (2.0 g, 95%, average size 800 nm, H.C. Starck, Newton, MA, USA), TTIP (2 mL, 97%, titanium(IV) isopropoxide, Sigma-Aldrich, St. Louis, MO, USA), anhydrous hexane (15 mL, 95%, Sigma-Aldrich), and tungsten carbide balls (40.0 g). The dry milling process was the same as that of the wet milling process except the use of hexane solvent. A nitrogen atmosphere was maintained in the milling jar during the milling process. TiO_2_-coated boron particles were obtained by varying the operation time from 1 to 8 h. During the milling process, TTIP was added to the milling jar at 2 h intervals. Post-processing was performed immediately after completion of the milling process to convert the suspension into a dried powder, and to remove physisorbed residues on the boron particles. This post-processing involved washing the boron particles in an ultrasonic bath following the addition of methanol (99.9%, Sigma-Aldrich), centrifugation at 3500 rpm (MF 80, Hanil Inc., Inchon, Korea), and drying in an oven at 100 °C for 1 h.

### 3.2. Characterization of the Coated Boron Particles

The characteristics of the TiO_2_-coated boron particles prepared via ball milling were determined using SEM to observe the changes in particle morphology with milling time, XPS, FT-IR, and Cs-corrected scanning transmission electron microscopy (Cs-STEM) equipped with an EDX spectrometer to examine the chemical bonding state and composition, and XRD and Cs-STEM to analyze the crystallographic properties of un-milled and milled boron particles. Finally, NPSA (NANOPHOX, Sympatec-GmbH Inc., Clausthal-Zellerfeld, Germany) was used to investigate the effects of milling operation time on particle size. The boron samples were prepared for SEM and XPS by attaching the particles to a carbon tape. SEM images were obtained on a JEOL JSM-7000F (JEOL Inc., Peabody, MA, USA) operated at a beam energy of 0.5–30 kV. XPS measurements were performed on a MultiLab 2000 (Thermo Scientific, West Palm Beach, FL, USA) equipped with a high performance Al Kα X-ray source. The base pressure of the XPS system was 5 × 10^−10^ Torr, and during data collection the XPS chamber pressure was ~5 × 10^−9^ Torr. High resolution spectra were collected using a pass energy of 20 eV with 0.1 eV/step. XPS sputtering was performed over 0, 60, or 240 s using a 3 kV and 1 μA beam rastered across a 2 × 2 mm area of the sample surface. Advantage 4.45 software was employed for XPS data analysis. For spectral curve fitting, the combination ratio of Lorentzian to Gaussian functions and the full width at half maximum (FWHM) fit parameter were fixed at 30% and 2.0 (±0.2) eV, respectively. Binding energies (BEs) were referenced to the adventitious C 1s peak at 284.5 eV. FT-IR spectra were measured using a FTS-175C FT-IR spectrometer (Bio-Rad Laboratories Inc., Hercules, CA, USA) and the spectra were recorded ~100 times between 400 and 2000 cm^−1^ to improve the S/N ratio. XRD patterns of the milled boron particles were obtained in the 2θ range from 10° to 80° using a Bruker D8 Advance (Bruker, Madison, WI, USA) X-ray powder diffractometer using a Cu Kα radiation (40 kV and 40 mA) source and Linxeye 1-D detector (Bruker) with an acquisition time of 5 s/step, and a step width of 0.02°. For Cs-STEM analysis, the particles were fully dispersed in ethanol by ultrasonication over 1 h. A few drops of the dispersion were deposited onto a lacey carbon-coated Cu grid for imaging. Cs-STEM (JEM-ARM200F, JEOL Inc.) with EDX (Bruker Quantax 400, Bruker) was operated at 200 kV to obtain the STEM images. Finally, NPSA based on photon cross-correlation spectroscopy was employed to obtain the size distribution of particles suspended in methanol.

### 3.3. Photoelectrochemical Measurements

LSV, CV, I-t amperometry, and electrochemical impedance were measured using a three-electrode cell configuration (Ag/AgCl reference electrode, Pt wire counter electrode, and sample working electrode) in a 0.1 M Na_2_SO_4_ electrolyte using a CHI660D electrochemical work station (CH Instruments, Austin, TX, USA). For the preparation of the working electrode, the powder samples were first dispersed in ethyl cellulose and α-terpineol-containing ethanol solution, pasted on a Si substrate (1 cm × 1 cm), and fully dried in a vacuum oven. The powder covered area was electrically connected with a Cu wire and the uncovered area was fully protected by silicon epoxy. For the I-t amperometry under cyclic ON and OFF light exposure, we used wavelengths of 365 (Mic-LED-365, Prizmatix, Givat-Shmuel, Israel) and 532 nm (SDL-532-200T, Shanghai Dream Lasers, Shanghai, China) for UV and visible light, respectively. Air was removed from the electrolyte by bubbling nitrogen gas through the solution for 30 min prior to electrochemical measurements. The working electrode was prepared by the addition of α-terpineol (0.25 mL) to a mixture of the desired sample (50 mg) in ethanol (2 mL). Following sonication of the resulting mixture for the desired time, ethanol (1 mL) and ethyl cellulose (0.05 mL) were added, and the mixture was subjected to sonication once again. After the desired sonication time, a further portion of ethanol (2 mL) was added, and the final mixture was sonicated a final time to provide a uniform mixture. The resulting solution was allowed to stand in a vacuum oven (40 °C) until the solvent evaporated to yield a gel, which was subsequently pasted on a Si substrate and dried in a vacuum oven at 70 °C. Finally, the electrode was prepared using Ag paste at the electric junctions combined with Cu wire.

## 4. Conclusions

In summary, we have described the development of a novel method to produce TiO_2_-coated boron particles via a wet ball milling process. Upon increasing the milling operation time, the average size of the wet milled boron particles decreased, with a reduction from 600 to 312 nm being recorded following wet milling for 8 h. Longer milling times also contributed to a narrower particle size distribution. Based on XPS, TEM-EDX, and FT-IR results, it was confirmed that the boron particles were coated with TiO_2_. Based on the XPS results, the overlayer Ti showed an oxidation state of 4+, attributed to TiO_2_. In addition, EDX results confirmed a coating thickness of thinner than 10 nm on a 150 nm-diameter wet milled particle. Furthermore, FT-IR results showed that as the milling operation time increased, the intensities of the TiO_2_ and B–O peaks at 540 cm^−1^ and 1300–1400 cm^−1^ increased, indicating the formation of a B–O–Ti species on the boron particles. In terms of the photoelectrochemical measurements, an HER current density of 250 μA was obtained at a potential of 0.5 V for the B@Ti ball milled for 2 h, with a low HER onset close to 0.0 V being observed. Moreover, an OER current density of 220 μA was obtained at a potential of 1.0 V. Thus, based on the current results, we could confirm that the TiO_2_-coated boron particles produced via a ball milling method exhibited potential for application in photoelectrochemical HERs and OERs.

## Figures and Tables

**Table 1:** Summary of atomic compositions (%) of wet milled boron particles at various milling times.

Atom	1 h	2 h	4 h	6 h	8 h
B	95.5	96.29	92.86	93.27	92.75
O	4.56	3.12	6.03	4.56	4.85
Ti	0.0	0.0	0.15	0.21	0.35
W	0.40	0.59	0.96	1.97	2.05
Total	100	100	100	100	100

**Table 2:** Summary of surface atomic compositions of the un-milled and wet milled boron particles at various sputtering times.

	Un-Milled Boron Sputter Time (s)			4 h Wet Milled Boron Sputter Time (s)			8 h Wet Milled Boron Sputter Time (s)		
Peaks	0	60	240	0	60	240	0	60	240
B 1s	53.92	71.22	79.45	47.42	61.21	73.02	34.16	44.74	68.49
C 1s	19.27	12.38	6.25	21.16	12.32	6.1	22.23	19.42	9.56
O 1s	23.5	13.35	13.08	25.1	20.84	16.15	33.37	26.18	16.16
Ti 2p	-	-	-	1.42	1.01	0.99	3.59	2.17	1.04
W 4f	-	-	-	1.47	1.88	2.34	2.17	3.58	3.26
F 1s	3.31	3.05	1.22	1.98	1.69	0.65	1.85	1.48	0.57
Co 2p	-	-	-	1.44	1.04	0.75	2.63	2.45	0.91
Total	100	100	100	100	100	100	100	100	100
